# Atlantification drives recent strengthening of the Arctic overturning circulation

**DOI:** 10.1126/sciadv.adu1794

**Published:** 2025-07-11

**Authors:** Marius Årthun, Ailin Brakstad, Jakob Dörr, Helen L. Johnson, Carlo Mans, Stefanie Semper, Kjetil Våge

**Affiliations:** ^1^ Geophysical Institute, University of Bergen, and Bjerknes Centre for Climate Research, Bergen, Norway.; ^2^ Department of Earth Sciences, University of Oxford, UK.

## Abstract

The Arctic Ocean is the northern terminus of the Atlantic Meridional Overturning Circulation (AMOC), whose dense water masses are key to the global ocean circulation. The Arctic climate is rapidly changing, and it is not known how the Arctic Ocean overturning circulation is responding. Here, we use a high-resolution ocean reanalysis, corroborated by observations, to show that a poleward expansion of warm Atlantic waters and corresponding sea-ice loss, a phenomenon referred to as an “Atlantification” of the Arctic, has caused a poleward shift of the dense water source regions in recent decades (1993–2020). This is manifested in enhanced surface water mass transformation in the Arctic Ocean, compensating for a reduction in the Nordic Seas. The associated strengthening of the Arctic Ocean overturning circulation has ensured that the transport of dense overflow waters across the Greenland-Scotland Ridge to the AMOC’s lower limb has remained stable. Our results thus provide evidence for a resilient northern overturning circulation in a warming climate.

## Teaser

A less ice-covered Arctic shifts dense water formation northward, strengthening the Arctic Ocean overturning circulation.

## INTRODUCTION

**Fig. 1.**
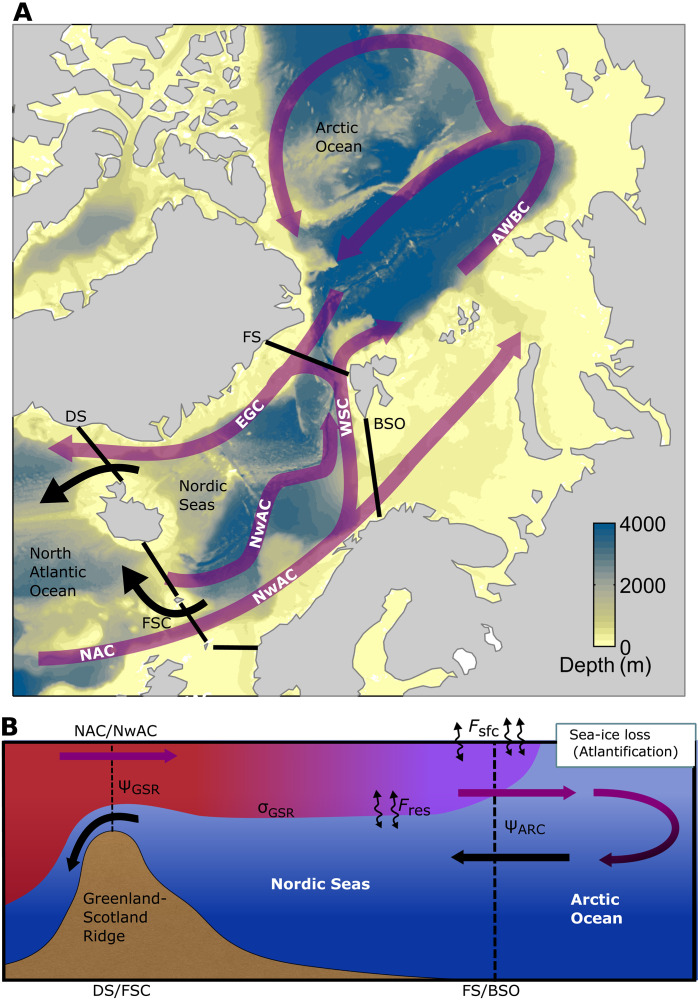
The overturning circulation in the Nordic Seas and Arctic Ocean. (**A**) Horizontal view of the ocean circulation in the Nordic Seas and Arctic Ocean. Warm Atlantic Water (AW) is transported northward by the North Atlantic Current (NAC), Norwegian Atlantic Current (NwAC), and West Spitsbergen Current (WSC). The AW enters the Arctic Ocean through the Barents Sea Opening (BSO) and Fram Strait (FS) and flows along the Atlantic Water Boundary Current (AWBC), before returning southward in the East Greenland Current (EGC). Cold, dense overflows (black arrows) exit the Nordic Seas through Denmark Strait (DS) and Faroe-Shetland Channel (FSC). The background color shows bottom depth in GLORYS12. (**B**) Vertical view of the overturning circulation in the Nordic Seas and Arctic Ocean. The terms used in the isopycnal volume budget used to quantify overturning changes are included (see Materials and Methods for a detailed description).

The Atlantic Meridional Overturning Circulation (AMOC), carrying warm, salty water to high latitudes, is a key component of the global ocean circulation with profound impacts on climate (*1*). To sustain the AMOC, dense water formation at northern high latitudes is a requirement (*2*). The Nordic Seas and Arctic Ocean (Fig. 1) are key regions of dense water formation (*3*–*5*). Here, substantial water mass transformation takes place as warm Atlantic Water (AW) enters the Nordic Seas at the Greenland-Scotland Ridge (GSR) and continues to the Arctic Ocean through the Barents Sea (*6*) and Fram Strait (*7*) and eventually forms a boundary current that encircles the Arctic Ocean (*8*, *9*). Along its pathways through the Nordic Seas and the Arctic Ocean, the AW experiences cooling and freshening by heat and freshwater exchanges with the atmosphere and by mixing with sea ice meltwater and fresher shelf waters (*10*, *11*). The transformed AW returns to the North Atlantic as dense overflow waters across the GSR that contribute substantially to the mean state and variability of the lower limb of the AMOC (*12*, *13*).

The Nordic Seas and Arctic Ocean are now experiencing amplified climate change, manifested in loss of sea ice (*14*) and ocean warming rates higher than the global average (*15*, *16*). In the Nordic Seas, this has reduced the cooling of the AW throughflow (*17*, *18*) and decreased the intensity of open-ocean convection (*19*, *20*). In contrast, an encroaching “Atlantification” from the expansion of Atlantic waters into the Arctic Ocean (*6*, *21*) has been accompanied by a recent sea-ice retreat that has partially uncovered the boundary current, leaving it exposed to the atmosphere in winter, which allows for further modification of the AW during transit (*22*–*24*). As the Atlantification of the Arctic continues, enhanced water mass transformation might, therefore, be expected, possibly strengthening the Arctic overturning circulation, which starkly contrasts the anticipated weakening of the North Atlantic overturning circulation (*25*, *26*).

Some models support a possible strengthening of the Arctic overturning circulation (*27*, *28*). Still, in those models, a strengthened Arctic overturning circulation only occurs through the onset of deep convection in the Arctic under strong warming and permanently ice-free summers. Other models show no emergence of deep convection in a warmer future Arctic (*29*). Such projections are based on coarse resolution climate models that do not resolve the transformation of AW along the boundary currents in the Nordic Seas and Arctic Ocean. It is, therefore, not known how the Arctic overturning circulation will respond to the ongoing Atlantification and associated changes in AW modification. As Arctic overturning occupies a key role in setting the strength and structure of the North Atlantic overturning circulation (*12*, *30*, *31*), this is a particularly important knowledge gap.

Here, we use the high-resolution (^1^/_12_°) ocean reanalysis GLORYS12 (*32*), corroborated by observations and other reanalyses, to show that the Arctic overturning circulation has increased in recent decades (1993–2020). This increase has compensated for reduced dense water formation in the Nordic Seas, leading to a stable supply of dense overflow waters to the North Atlantic Ocean. We thus demonstrate that a strengthened Arctic overturning circulation is not a potential feature of the future, but an ongoing consequence of Arctic Atlantification.

## RESULTS

### Reduced dense water formation in the Nordic Seas compensated for by increased Arctic overturning

We mainly use the high-resolution ocean reanalysis GLORYS12 to investigate potential changes in the Nordic Seas and Arctic overturning circulation during recent decades. Previous studies have shown that GLORYS12 compares well with observations in the Nordic Seas, Arctic Ocean, and subpolar North Atlantic (*18*, *33*–*36*). Specifically, the exchanges across the GSR are well simulated (*36*), including the dense overflow through the Faroe-Shetland Channel (FSC) (*34*). A comparison between GLORYS12 and available historical velocity observations demonstrates that the circulation within the Nordic Seas is also realistic (*18*). This is further corroborated by accurate AW transports in GLORYS12 through the Barents Sea Opening (BSO; fig. S1) and upstream in the NwAC (Svinøy section; fig. S2), although observed short-term (monthly) variations are not always reproduced by GLORYS12. The density and velocity fields across Fram Strait and the BSO are also in good agreement with observations (fig. S3). The southward velocities in western Fram Strait are somewhat weaker in GLORYS12, consistent with an underestimated liquid freshwater transport (*37*), but the net volume transport through Fram Strait is, nevertheless, similar to observations (*32*). GLORYS12 is thus an appropriate product to study dense water dynamics in the Nordic Seas.

We use a water mass transformation framework to determine the processes setting the mean strength and variability in the Nordic Seas and Arctic Ocean overturning circulation (see Materials and Methods) (*38*–*41*). Considering dense water formation within the Nordic Seas, bounded by the GSR and the Arctic gateways (Fram Strait and the BSO; Fig. 1), this framework allows us to establish the balance between dense waters produced within the basin and dense waters being exported across the boundaries. Here, we calculate the overturning streamfunction in density-space ($\psi_{\sigma }$ ; Materials and Methods) across the GSR and the two Arctic gateways (collectively referred to as ARC) and relate the overturning transport (dense water export) at different density levels to water mass transformation by surface buoyancy fluxes ( $F_{\text{sfc}}$ ) and interior diapycnal mixing ( $F_{\text{res}}$ ) within the Nordic Seas.

**Fig. 2.**
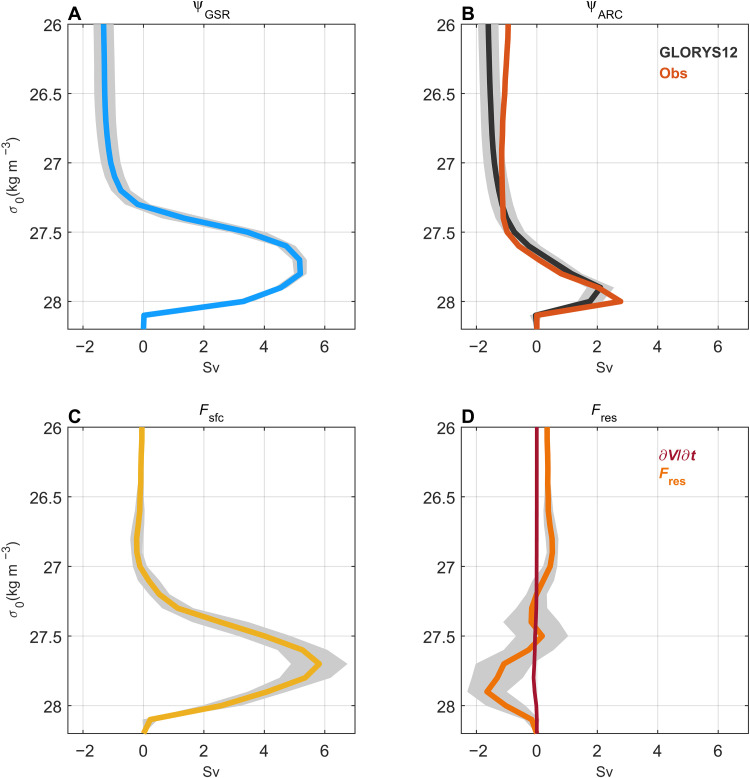
Time-mean overturning circulation and water mass transformation. (**A** and **B**) Streamfunctions of density-space overturning strength at the GSR ( $\psi_{\text{GSR}}$ ) and the Arctic gateways (Fram Strait and BSO; $\psi_{\text{ARC}}$ ) in GLORYS12 between 1993 and 2020. (**C**) Diapycnal water mass transformation from surface buoyancy forcing in the Nordic Seas ( $F_{\text{sfc}}$ ). (**D**) Volume change ( ∂V/∂t ) and residual water mass transformation ( $F_{\text{res}}$ ). Gray shading is the interannual standard deviation. The observation-based estimate in (B) is from (*46*) and is based on data from 2005 to 2009. As the mean volume tendency is close to zero, it follows that $\psi_{\text{GSR}}-\psi_{\text{ARC}}\sim F_{\text{sfc}}+F_{\text{res}}$ (Materials and Methods). Sv, sverdrup.

In GLORYS12, the time-mean density-space overturning across the GSR ( $\psi_{\sigma {\;|}_{\text{GSR}}}$; Fig. 2A) has a maximum of 5.2 sverdrup (1 sverdrup ≡ 10^6^ m^3^ s^−1^). The density associated with this maximum ( $\sigma_{\text{GSR}}$ = 27.8 kg m^−3^) corresponds to the density used to define dense overflow waters in observations (*3*). The maximum overturning strength is also referred to as the volume transport of the lower limb of the overturning circulation. The overturning across the Arctic gateways ( $\psi_{\sigma {\;|}_{\text{ARC}}}$; Fig. 2B) is smaller than across the GSR but still substantial (2.1 sverdrup at $\sigma_{\text{ARC}}$ = 27.9 kg m^−3^). The surface-forced water mass transformation over the Nordic Seas ( $F_{\text{sfc}}$; Fig. 2C) closely resembles the overturning circulation across the GSR, demonstrating that surface buoyancy forcing is a major source of water mass transformation in the Nordic Seas (*42*, *43*). Diapycnal mixing (residual term; $F_{\text{res}}$; Fig. 2D) also makes an important contribution, especially at higher densities. The predominantly negative values of the residual term indicate that diapycnal mixing acts to transform waters to lower density. An important contribution of mixing-driven water mass transformation in the Nordic Seas is consistent with observations (*44*, *45*) and simulations (*31*, *40*). The mean volume change (∂V/∂t ) is close to zero (Fig. 2D).

**Fig. 3.**
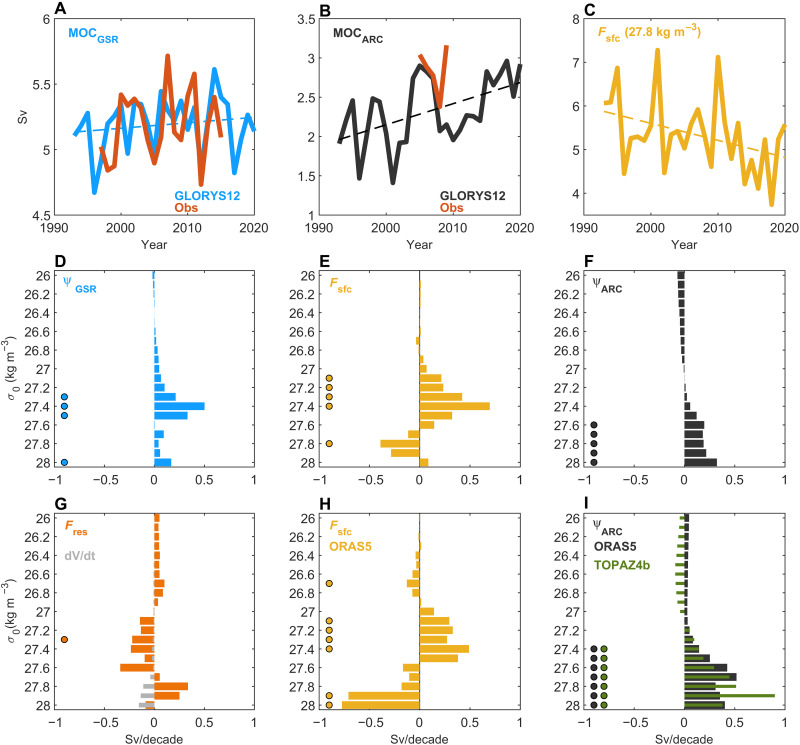
Overturning trends. Time series of overturning strength at (**A**) the GSR ( $\text{MO}C_{\text{GSR}}$ ) and (**B**) the Arctic gateways ( $\text{MO}C_{\text{ARC}}$ ), and (**C**) time series of surface-forced water mass transformation in the Nordic Seas ( $F_{\text{sfc}}$ ) at $\sigma_{o}$ = 27.8 kg m^−3^. Observations in (A) are from the AtlantOS consortium (*63*), while the observation-based estimate in (B) is from (*46*). (**D** to **I**) Linear trends (in sverdrup decade^−1^, 1993–2020) at different density levels in the overturning strength at GSR ( $\psi_{\text{GSR}}$ ) and across the Arctic gateways ( $\psi_{\text{ARC}}$ ), in surface water mass transformation ( $F_{\text{sfc}}$ ), volume changes ( ∂V/∂t ), and in the residual overturning ( $F_{\text{res}}$ ). Trends in (D) to (G) are from GLORYS12, while (H) and (I) are based on ORAS5 and TOPAZ4b. Dots indicate significant trends at the 95% confidence level. Sv, sverdrup.

During the period 1993–2020, the strength of the overturning circulation at the GSR ( $\text{MO}C_{\text{GSR}}$ ; defined as the overturning strength at $\sigma_{\text{GSR}}$ = 27.8 kg m^−3^; Materials and Methods) shows pronounced interannual variability but little long-term change (Fig. 3A). In contrast, both the Arctic overturning circulation ( $\text{MO}C_{\text{ARC}}$ ) and the surface-forced water mass transformation within the Nordic Seas display clear and significant long-term trends (Fig. 3, B and C). The contribution from diapycnal mixing shows little long-term change (fig. S4). This implies that a stable overturning circulation at the GSR (Fig. 3A) has been maintained by an increasing overturning across the Arctic gateways (Fig. 3B), which has compensated for decreasing dense water formation by surface buoyancy fluxes over the Nordic Seas (Fig. 3C).

Considering all density levels reveals that there are different trends for lighter and denser water masses. At the GSR, there are significant positive trends in the upper limb overturning (i.e., for densities lower than 27.8 kg m^−3^; Fig. 3D). The strengthening of the upper limb overturning circulation at the GSR is associated with increased surface transformation at these density levels but reduced transformation of denser waters (Fig. 3E). These results suggest that surface buoyancy forcing in the Nordic Seas has not been sufficient to transform the increased upper limb transport across the GSR into lower-limb overturning (overflow) waters during recent decades. The decreasing light-to-dense surface water mass transformation in the Nordic Seas has, however, been compensated for by increased export of dense waters from the Arctic Ocean (Fig. 3F) and less dense-to-light water transformation by diapycnal mixing (Fig. 3G, positive trends at overflow densities imply less negative values). The trends in the storage rate ( ∂V/∂t ) of dense waters are generally small and not significant (Fig. 3G), indicating a long-term balance between water mass transformation and export. The transport of dense water between the Nordic Seas and the subpolar North Atlantic across the GSR has, therefore, remained stable (Fig. 3D). Consistent with these results, two other ocean reanalyses (ORAS5 and TOPAZ4b; their time-mean Arctic overturning streamfunctions are shown in fig. S5) also show reduced surface dense water formation in the Nordic Seas (Fig. 3H) and enhanced Arctic overturning circulation between 1993 and 2020 (Fig. 3I).

**Fig. 4.**
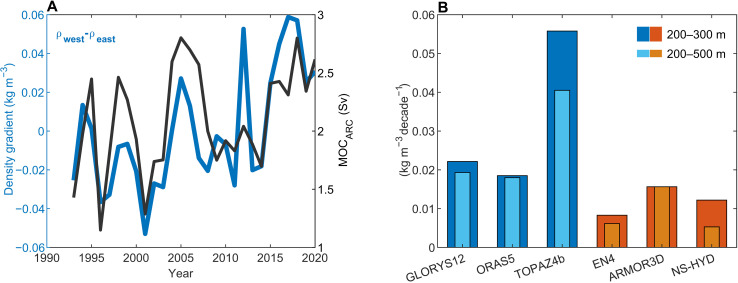
Observed and modeled changes in Fram Strait density gradient. (**A**) Zonal (west-east) density gradient anomalies (relative to 1993–2020 mean) across Fram Strait at 79°N. The gradient is calculated between 2°W to 5°W ( $\rho_{\text{west}}$ ) and 7°E to 11°E ( $\rho_{\text{east}}$ ) at 200 to 300 m. Sv, sverdrup. (**B**) Trend in the zonal density gradient across Fram Strait (200 to 300 m and 200 to 500 m) between 1993 and 2020 in ocean reanalyses (GLORYS12, ORAS5, and TOPAZ4b) and purely observation-based products (EN4, ARMOR3D, and NS-HYD; Materials and Methods).

An observation-based estimate of the Arctic overturning strength exists (*46*) but only covers the period 2005–2009 (if considering annual averages) and, therefore, cannot be used to evaluate the long-term trend in the reanalyses. As an observation-based proxy for the Arctic overturning strength, we instead use the subsurface zonal density gradient across Fram Strait, which captures the variability and trend in Arctic overturning in GLORYS12 [Fig. 4A; correlation coefficient (*r*) = 0.70]. We chose the specific depth ranges (200 to 300 m and 200 to 500 m) used to calculate the density gradient to capture the density differences between the inflowing AW in the east and the outflowing dense intermediate waters in the west ( $\sigma_{0}$ = 27.9 to 28.0 kg m^−3^; fig. S3). Consistent with a strengthened Arctic overturning, all three ocean reanalyses and three observation-based products (Materials and Methods) show a strengthened zonal density gradient in Fram Strait between 1993 and 2020 (Fig. 4B).

### Poleward shift in dense water formation

**Fig. 5.**
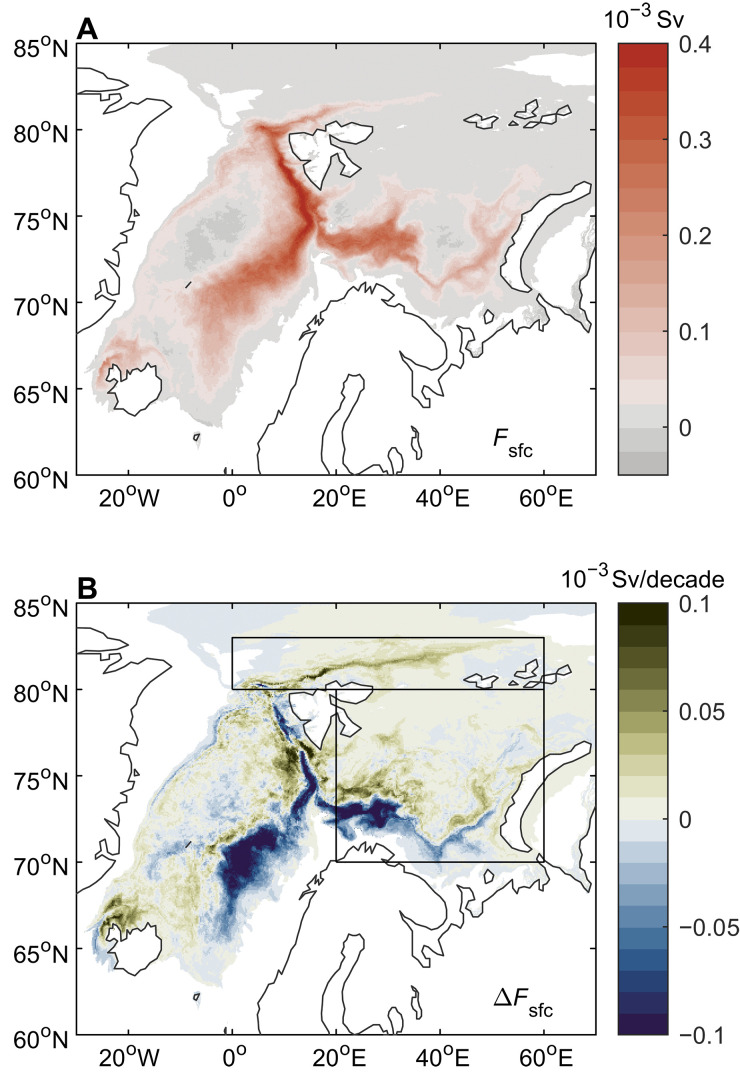
Poleward shift in dense water transformation. (**A**) Time-mean spatial pattern of surface-forced overturning ( $F_{\text{sfc}}$ ) at $\sigma_{0}$ = 27.8 kg m^−3^ in GLORYS12. Positive values correspond to densification to this isopycnal. (**B**) Linear trend of surface-forced overturning ( $\Delta F_{\text{sfc}}$ ) at $\sigma_{0}$ = 27.8 kg m^−3^ between 1993 and 2020. Sv, sverdrup.

To further understand the contrasting changes in the Arctic overturning (Fig. 3B) and surface-forced water mass transformation over the Nordic Seas (Fig. 3C), Fig. 5 shows maps of the mean and changing surface-forced water mass transformation across the density surface $\sigma_{0}$ = 27.8 kg m^−3^. The reduced surface transformation of waters at this density level in the Nordic Seas is predominantly a result of less transformation along the outer branch of the Norwegian Atlantic Current (NwAC) and along the West Spitsbergen Current (WSC). Along the NwAC, the decrease in surface-forced water mass transformation is predominantly a result of a reduction in the outcropping area of the 27.8 kg m^−3^ isopycnal and not because of decreased surface buoyancy fluxes (fig. S6; Materials and Methods). We note, however, that these two factors are somewhat interdependent, i.e., surface heat loss impacts surface temperature and, hence, density. Surface buoyancy fluxes are, on the other hand, important to water mass transformation changes along the WSC, which is also the region with the largest reduction in surface heat fluxes (fig. S7A). The spatial pattern of surface-forced water mass transformation trends at $\sigma_{0}$ = 27.9 kg m^−3^ (fig. S8) looks similar to that for 27.8 kg m^−3^. For 28.0 kg m^−3^, there is reduced transformation along the WSC and its westward recirculation and enhanced transformation in the Greenland Sea (figs. S9 and S10), resulting in a small (not significant) positive overall trend for the Nordic Seas (Fig. 3E). This enhanced transformation of the densest water is still relatively small compared with the reduction at $\sigma_{0}$ = 27.8 to 27.9 kg m^−3^.

**Fig. 6.**
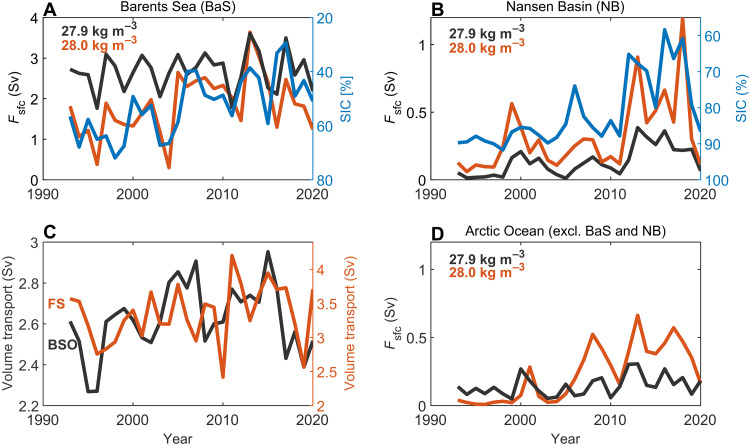
Enhanced water mass transformation in the Arctic Ocean. Time series of surface-forced water mass transformation in (**A**) the Barents Sea, (**B**) the Nansen Basin (the two regions are indicated in Fig. 5), and (**D**) the rest of the Arctic Ocean (bounded by the Bering Strait and Davis Strait; fig. S11). The mean sea-ice concentration (SIC) is also shown (inverted) in (A) and (B). (**C**) Time series of AW volume transport through Fram Strait (FS; *T* > 2°C, *S* > 34.8; red line) and the BSO (*T* > 3°C, *S* > 34.8; black line). Sv, sverdrup.

While surface transformation of dense waters has reduced in the Nordic Seas, we can see increased transformation in the northern Barents Sea and in the Nansen Basin, north of Svalbard (Fig. 5B). Averaging over the whole Barents Sea, we find no net change in transformation of waters across the density surface $\sigma_{0}$ = 27.8 kg m^−3^, while there is an increase at higher densities (Fig. 6A). North of Svalbard, increased water mass transformation is dominated by a step change between 2011 and 2012 (Fig. 6B). The increase in water mass transformation north of Svalbard and in the northern Barents Sea is mainly a result of surface density changes (larger isopycnal outcrop area; figs. S6 and S10) associated with sea-ice loss (Fig. 6, A and B) (*6*, *14*), enhanced surface heat loss (fig. S7A) (*23*, *47*), and increased winter convection (fig. S7B) (*24*). The enhanced surface-forced water mass transformation is not associated with an increased poleward flow of AW, which shows little change for both the BSO and Fram Strait (Fig. 6C).

The increase in surface-forced transformation of dense waters in the Arctic Ocean during recent decades (1993–2020) together with the concomitant reduction in water mass transformation in the Nordic Seas demonstrates that there has been a poleward shift in the source region of dense waters that maintain the lower limb of the Nordic Seas and North Atlantic overturning circulations. The increased surface-forced water mass transformation in the Barents Sea [ $\Delta F_{\text{sfc}}$ (27.9 kg m^−3^) = 0.2 sverdrup based on the linear trend between 1993 and 2020] and north of Svalbard (0.4 sverdrup) is sufficient to explain the increase in Arctic overturning ( $\mathrm{\Delta MO}C_{\text{ARC}}$ = 0.6 sverdrup; Fig. 2B). There is little surface-forced transformation of dense waters elsewhere in the Arctic Ocean (Fig. 6D and fig. S11) and no trend in dense-water storage. As exchanges between the Arctic Ocean and other gateways (most notably, Bering Strait and Davis Strait) do not contribute to the dense overturning circulation in the Arctic (*46*), this indicates a balance between the increasing (long-term trend) transformation of dense waters in the Barents Sea and Nansen Basin and export through Fram Strait/BSO. Note that such a balance does not necessarily hold on shorter (interannual) timescales (*39*).

### Warming of dense waters

**Fig. 7.**
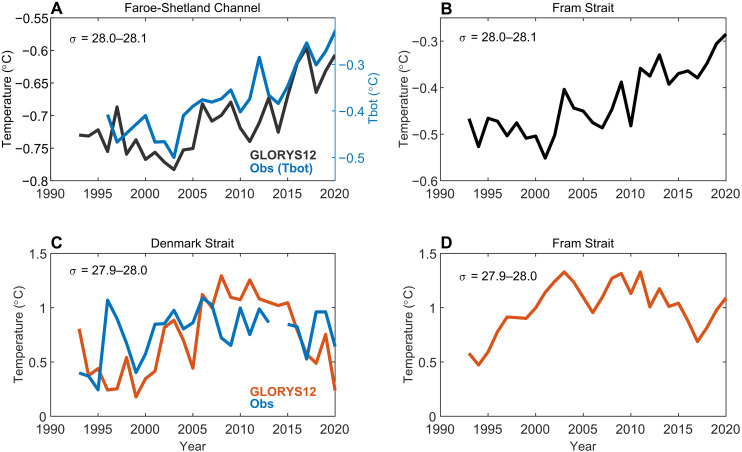
Dense water temperatures. Transport-weighted temperatures for different density intervals in (**A**) the FSC, (**B** and **D**) Fram Strait, and (**C**) Denmark Strait. The transport-weighted temperatures only consider southward-flowing waters. Blue line in (A) is bottom temperature (Tbot) in observations (*48*). Observations from Denmark Strait (C) are based on the hydrographic database NS-HYD (*50*). Note the different *y* axes.

Although compensating changes in dense water formation in the Nordic Seas versus the Arctic Ocean have kept the strength of the overturning circulation across the GSR unchanged over recent decades, the properties of the overflow waters have not remained constant. In the FSC, the overflow waters are warming (*48*). This warming trend is also seen in GLORYS12 (Fig. 7A) with similar magnitude (0.08°C/decade and 0.10°C/decade between 2000 and 2020 for GLORYS12 and observations, respectively). In support of an upstream source of the warming of FSC overflow waters, dense waters in Fram Strait ( $\sigma_{0}$ = 28.0 to 28.1 kg m^−3^; Fig. 7B) show comparable warming (0.07°C/decade). The interannual temperature variability of the dense outflow waters in Fram Strait is also similar to that in the FSC (*r* = 0.52 for detrended data when Fram Strait leads by 4 years), with a lag consistent with the transit time of observed temperature anomalies (*49*). Consistent with observations (*48*), the warming of the FSC overflow waters is accompanied by higher salinities, resulting in no change to the density. In contrast to the FSC, overflow temperatures in Denmark Strait from both observations and GLORYS12 show no significant trend (Fig. 7C). There is, however, pronounced interannual and decadal variability, the latter characterized by increasing temperatures in the late 1990s and 2000s and decreasing temperatures during the recent decade (2010s). Similar variability is also found for dense waters in Fram Strait with $\sigma_{0}$ = 27.9 to 28.0 kg m^−3^ (*r* = 0.60 when Fram Strait leads by 3 years; Fig. 7D).

The density and temperature of the lighter dense waters in Fram Strait (Fig. 7D) suggest that these have their source in Arctic Atlantic Waters, i.e., AW that has been modified within the Arctic Ocean (*45*). AW recirculating in Fram Strait is characterized by higher temperatures. The denser waters (Fig. 7B) have properties in line with upper Polar Deep Water, which is the main intermediate water mass of the central Arctic Ocean (*45*). Hence, although we have not assessed the impact of variable dense water formation in other regions, e.g., the Greenland Sea (*20*, *50*), these results support an important impact of Arctic Ocean dense waters on overflow temperatures downstream at the GSR.

## DISCUSSION

This study has examined the overturning circulation in the Nordic Seas and Arctic Ocean and its drivers using a water mass transformation framework (Materials and Methods) (*38*–*41*). We demonstrate that there has been a recent strengthening of the Arctic overturning circulation. Using a high-resolution (^1^/_12_°) ocean reanalysis (GLORYS12), we show that, while dense water formation from surface forcing in the Nordic Seas has decreased over the recent decades (1993–2020), the formation and export of Arctic Ocean dense waters have increased. This poleward shift of the northern source region for the AMOC is related to a retreating sea-ice cover along the AW pathways north of Svalbard and in the Barents Sea (*23*) and, hence, is intimately linked to the ongoing Atlantification of the Arctic (*6*, *21*). As a result of a strengthened Arctic overturning circulation, the supply of dense overflow waters from the Nordic Seas to the North Atlantic Ocean has remained stable.

Our analysis is mainly based on GLORYS12, which compares well to observations both in terms of water masses and transports in the Nordic Seas and Arctic Ocean (Figs. 2B and 3B and figs. S1 to S3) (*18*, *34*–*36*). A recent strengthening of the Arctic overturning circulation is also corroborated by two other ocean reanalyses (ORAS5 and TOPAZ4b; Fig. 3I) and by a strengthened zonal density gradient across Fram Strait in both reanalyses and observations (Fig. 4B). A common source of uncertainty for ocean reanalyses is the impact of data assimilation. This can introduce external sources of heat and mass that can lead to enhanced variability and spurious long-term trends (*51*). The favorable comparisons with observations nevertheless provide confidence in our results. The mechanism of a strengthened Arctic overturning circulation (increased surface water mass transformation in areas of sea-ice retreat) is also firmly rooted in observations (*22*, *23*).

The results presented here concern changes in the Nordic Seas and Arctic Ocean overturning circulation over recent decades, a period of rapid Arctic climate change. Climate model projections show a sustained incursion of Atlantic waters into the Eurasian Basin throughout the century (*16*, *47*, *52*), supporting a strengthened Arctic overturning circulation also in the future. The near-term trajectory of the Arctic overturning circulation will, however, also be determined by natural (internal) climate variability. In the Nordic Seas and Arctic Ocean, this is manifested in pronounced decadal variability in the poleward flow of AW (*52*, *53*) and, hence, in the extent of Arctic Atlantification.

The strengthening of the Arctic overturning, as demonstrated here, provides evidence for a resilient northern overturning circulation in a warming climate. The resulting steady supply of dense waters helps sustain the lower limb of the AMOC. Current generation climate models do not accurately resolve water mass transformation processes in the Arctic Ocean and, hence, diverge in their projections of Atlantification (*54*) and the emergence of new areas of deep convection (*29*). The stabilizing role of the Arctic Ocean in future AMOC projections could, therefore, be underestimated, and a better understanding of the Arctic overturning circulation and its representation in models is essential to constrain the future strength and structure of the AMOC.

## MATERIALS AND METHODS

### Ocean reanalyses

The main results in this study are based on the high-resolution Global Ocean Physics Reanalysis [hereafter, GLORYS12 (*32*)]. GLORYS12 uses the Nucleus for European Modeling of the Ocean (NEMO) ocean model and the LIM2 sea ice model with a horizontal resolution of ^1^/_12_° and 50 depth layers. A variety of in situ and satellite data, including sea level, surface and subsurface hydrography, sea ice concentration, and thickness, are assimilated using a reduced-order Kalman filter. At the surface, the ocean model is driven by the ECMWF ERA-Interim atmospheric reanalysis (*55*). Previous studies show that GLORYS12 compares well with observations in the Nordic Seas, Arctic Ocean, and subpolar North Atlantic (*18*, *33*–*36*). In this study, we provide further comparisons between GLORYS12 and observations (figs. S1 to S3).

In addition to GLORYS12, we also use data from ORAS5 (*56*). ORAS5 is also based on the NEMO model and has a horizontal resolution of ^1^/_4_° and 75 depth layers. ORAS5 performs well in the Nordic Seas (*43*) and Arctic Ocean (*53*, *57*). We also use data from the coupled ocean and sea ice data assimilation system TOPAZ4b (*58*, *59*). TOPAZ4b is based on the HYCOM ocean model and uses the ensemble Kalman filter to assimilate different types of ocean and sea ice observations. The model covers the North Atlantic and the Arctic with a horizontal resolution of 12 to 16 km.

### Observations

We use monthly fields of gridded temperature, salinity, and velocity across Fram Strait and the BSO between October 2004 and May 2010 (*46*) to evaluate the overturning streamfunction at the Arctic gateways and the hydrographic structure across Fram Strait and BSO in the ocean reanalyses. This dataset has been constructed using available moored instruments and hydrographic data together with a box inverse model (*46*). Given the use of an inverse model, we will refer to these data as “observation based.” We note that the moored instruments used in the box inverse model are not assimilated in the ocean reanalyses.

Observed transport of AW (*T* > 3°C) into the Barents Sea in GLORYS12 is evaluated on the basis of current meter moorings in the BSO (71.5°N to 73.5°N, 20°E) operated by the Institute of Marine Research, Norway (*60*). Monthly data are available from September 1997 until March 2017 (*61*). We also compare GLORYS12 with the observed current speed in the core of the Norwegian Atlantic Slope Current (Svinøy section; 62°48′N, 4°55′E, 100-m depth) since 1995 (*62*), with observed transports of overflow water ( $\sigma_{0}$ > 27.80 kg m^−3^) through the Faroe Bank Channel and Denmark Strait between 1997 and 2015 (*63*) and with observed overflow (bottom) temperatures from the Faroe Bank Channel (site FB) since 1995 (*48*). We use the hydrographic database from (*50*) that combines all available observations (Argo and shipboard measurements) from the Nordic Seas (here referred to as NS-HYD) to assess overflow temperatures in Denmark Strait. Temperature and salinity profiles in Denmark Strait were interpolated onto regular depth levels before calculating annual mean overflow ( $\sigma_{0}$ = 27.9 to 28.0 kg m^−3^) temperatures. Note that the dataset contains no observations from 2013 and 2014.

Temperature and salinity fields from NS-HYD (*50*), EN4 [EN4.2.2, (*64*)], and ARMOR3D (*65*) are analyzed to evaluate the long-term (1993–2020) trend in the zonal density gradient across Fram Strait. EN4 comprises available hydrographic data from ships and Argo floats interpolated on a 1° horizontal grid with 42 levels in the vertical. ARMOR3D also merges different sources of observations and provides monthly three-dimensional temperature and salinity fields with a horizontal resolution of 0.25° and 50 vertical levels. These observation-based products have been extensively used to investigate hydrographic variability in the Nordic Seas and North Atlantic (*33*, *40*, *66*, *67*).

### Calculation of overturning circulation

We calculate the isopycnal volume and buoyancy budget for the Nordic Seas following previous studies (*38*–*41*)

$$\frac{\partial V_{\sigma }}{\partial t}=M_{\sigma }-F_{\sigma }$$

where $\partial V_{\sigma }\;/\;\partial t$ is the temporal volume change (in sverdrup) of fluid denser than σ within the Nordic Seas, which is here bounded in the south by the GSR and in the north by Fram Strait (79°N) and the BSO (collectively denoted the Arctic gateways; ARC) (Fig. 1). The location of the Arctic gateways is based on the location of observational arrays and previous studies (*30*, *46*).

The overturning divergence, $M_{\sigma }$ , is defined as

$$M_{\sigma }=\psi_{\sigma {\;|}_{\text{GSR}}}-\psi_{\sigma {\;|}_{\text{ARC}}}$$

where $\psi_{\sigma }$ is the overturning streamfunction in density-space along a section and is calculated as (*34*)

$$\psi_{\sigma }=-\int_{\sigma_{\text{max}}}^{\sigma }\int_{x_{w}}^{x_{e}}v\left(x,\sigma \right)\;\mathrm{dx}\;d\sigma^{*}$$

where $x_{w}$ and $x_{e}$ denote the start and end of the sections, respectively; $\sigma_{\text{max}}$ is the maximum density; and v(x,σ) is the velocity perpendicular to the section (positive northward). The isopycnal volume budget is performed for a closed domain (closed mass budget), and no compensation is, therefore, applied to account for the small (<1 sverdrup) net transport through each section. We calculate time series of overturning strength in density-space across the GSR (MOC_GSR_; Fig. 3A) and the Arctic gateways (MOC_ARC_; Fig. 3B) based on the respective time-mean isopycnals of the maximum overturning streamfunction

$$\text{MO}C_{\text{GSR}}\left(t\right)=\psi_{\sigma {\;|}_{\text{GSR}}}\left(\sigma_{\text{GSR}},t\right)$$

$$\text{MO}C_{\text{ARC}}\left(t\right)=\psi_{\sigma {\;|}_{\text{ARC}}}\left(\sigma_{\text{ARC}},t\right)$$

where $\sigma_{\text{GSR}}$ = 27.8 kg m^−3^ and $\sigma_{\text{ARC}}$ = 27.9 kg m^−3^. Using time-variable $\sigma_{\text{GSR}}$ and $\sigma_{\text{ARC}}$ has no discernible impact on the overturning time series and trends.

$F_{\sigma }$ is the diapycnal transformation rate (in sverdrup) within the domain and is decomposed into two terms

$$F_{\sigma }=F_{\sigma }^{\text{sfc}}+F_{\sigma }^{\text{res}}$$

$F_{\sigma }^{\text{sfc}}$ , generally referred to as the surface-forced water mass transformation, is the diapycnal transformation driven by surface density flux acting over the area enclosed by outcropping isopycnal, σ , and is calculated as

$$F_{\sigma }^{\text{sfc}}=\frac{1}{\Delta \sigma }\int \left[-\frac{\alpha }{C_{p}}Q_{H}-\beta \frac{S}{1-S}Q_{\text{FW}}\right]\mathrm{dA}$$

Here, α is the thermal expansion coefficient, β is the haline contraction coefficient, $C_{p}$ is the specific heat capacity of seawater, $Q_{H}$ is the net surface heat flux into the ocean, S is surface salinity, and $Q_{\text{FW}}$ is the net freshwater flux into the ocean that includes evaporation, precipitation, sea ice melting/freezing and river runoff. The surface density flux is integrated over surface density outcrop regions *dA* for densities in the range σ−Δσ/2<σ<σ+Δσ/2 and Δσ = 0.1. If σ does not outcrop within the defined region in a given month, then $F_{\sigma }^{\text{sfc}}$ is set to zero. We obtain maps of $F_{\sigma }^{\text{sfc}}$ by accumulating the integrand over outcrops. $F_{\sigma }^{\text{sfc}}$ could not be calculated for TOPAZ4b as surface heat and freshwater fluxes were not available.

Surface-forced water mass transformation depends on both the ocean surface density field (*D*; setting the surface area, *dA*, covered by a particular isopycnal) and air-sea buoyancy fluxes (*Q*; a combination of $Q_{H}$ and $Q_{\text{FW}}$ ), that is, $F_{\sigma }^{\text{sfc}}\left(D,Q\right)$ (*68*). To assess the relative importance of *D* and *Q*, we also calculate $F_{\sigma }^{\text{sfc}}$ using (i) a combination of the variable surface density (*D*) and the mean (monthly climatology) heat and freshwater fluxes ( $\bar{Q}$ ), $F_{\sigma }^{\text{sfc}}\left(D,\bar{Q}\right)$ , and (ii) the mean surface density ( $\bar{D}$ ) and variable heat and freshwater fluxes (*Q*), $F_{\sigma }^{\text{sfc}}\left(\bar{D},Q\right)$.

After calculating all other terms in the isopycnal volume and buoyancy budget, we calculate $F_{\sigma }^{\text{res}}$ as a residual. This term is commonly interpreted as the transformation induced by interior diapycnal mixing (*39*–*41*) but also includes other unresolved processes, including the impact of data assimilation.

All calculations are performed on monthly data and then averaged into annual fields. Linear trends are calculated on the basis of the annual data by least squares fitting and their statistical significance assessed using a Student’s *t* test.
