# Supplementary material for: Atlantification drives recent strengthening of the Arctic overturning circulation
Source: Sci Adv. 2025 Jul 11;11(28):eadu1794. doi: 10.1126/sciadv.adu1794 (PMC12248306; doi:10.1126/sciadv.adu1794)
Supplement: Supplementary file 1 — Figs. S1 to S11 [file sciadv.adu1794_sm.pdf]

Science Advances
AAAS

Supplementary Materials for

Atlantification drives recent strengthening of the Arctic overturning circulation

Marius Årthun *et al.*

Corresponding author: Marius Årthun, marius.arthun@uib.no

Sci. Adv. **11**, eadu1794 (2025)
DOI: 10.1126/sciadv.adu1794

This PDF file includes:

Figs. S1 to S11

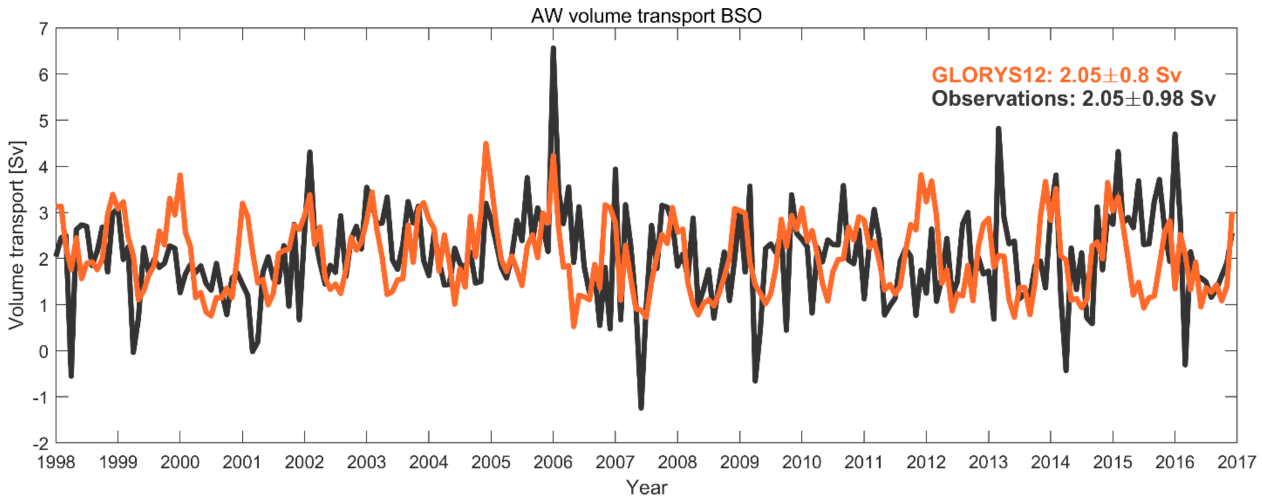

Fig. S1. Observed and simulated AW transport into the Barents Sea. Atlantic Water (temperature > 3°C) volume transport through the Barents Sea Opening (BSO; 71.5-73.5°N, 20°E) in observations (*60,61*) and GLORYS12. The mean values and standard deviations are provided. Note that, unlike in Fig. 6C, no salinity criterion is applied here as the observations do not cover the fresh coastal current.

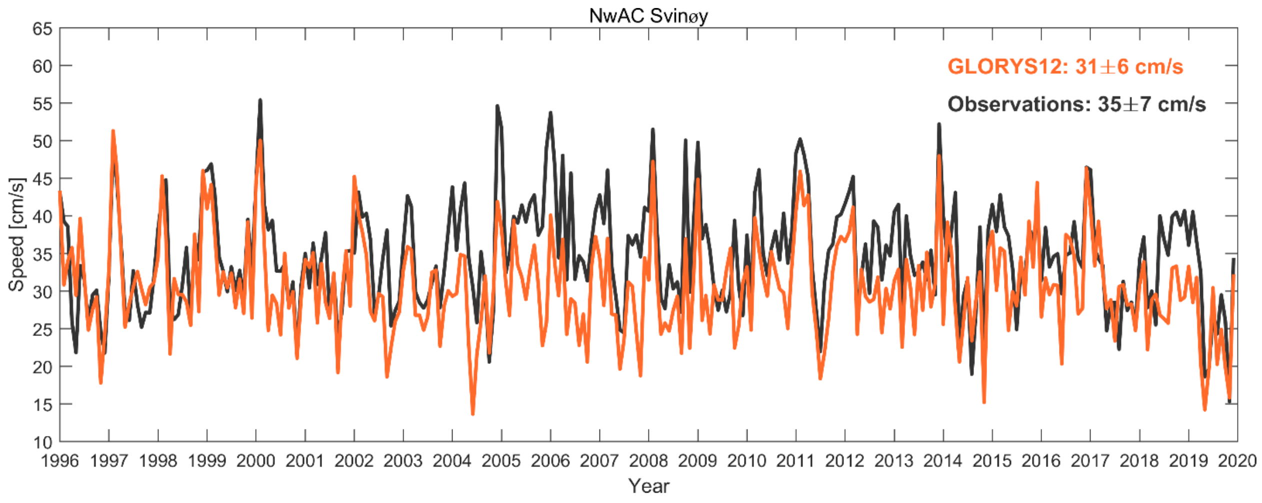

Fig. S2. Observed and simulated velocity of the Norwegian Atlantic Current. Current speed in the Norwegian Atlantic Current (NwAC), Svinøy section (62°48′N, 4°55′E, 100 m depth), in observations (*62*) and GLORYS12. The mean values and standard deviations are provided.

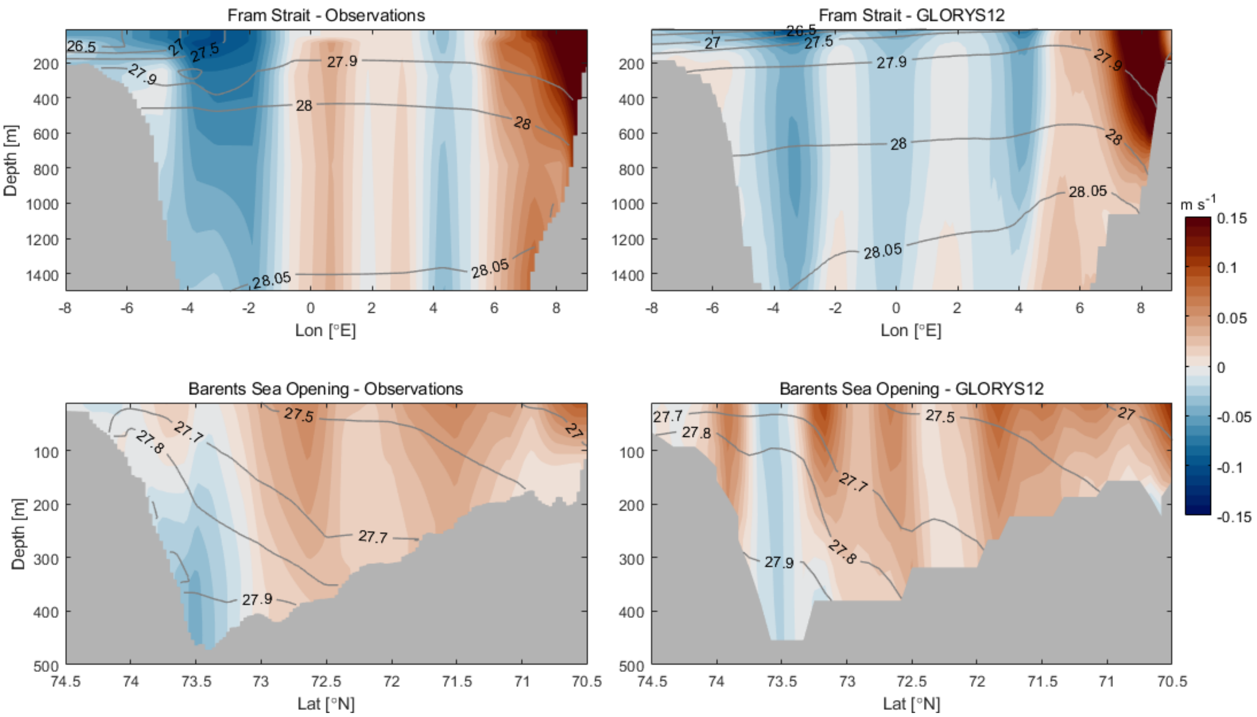

Fig. S3. Vertical sections of velocity and density at the entrance to the Arctic Ocean. Velocities (color; positive values indicate poleward flow) and density (contours) across Fram Strait and the Barents Sea Opening in observations (*46*) and GLORYS12 between 2005-2009.

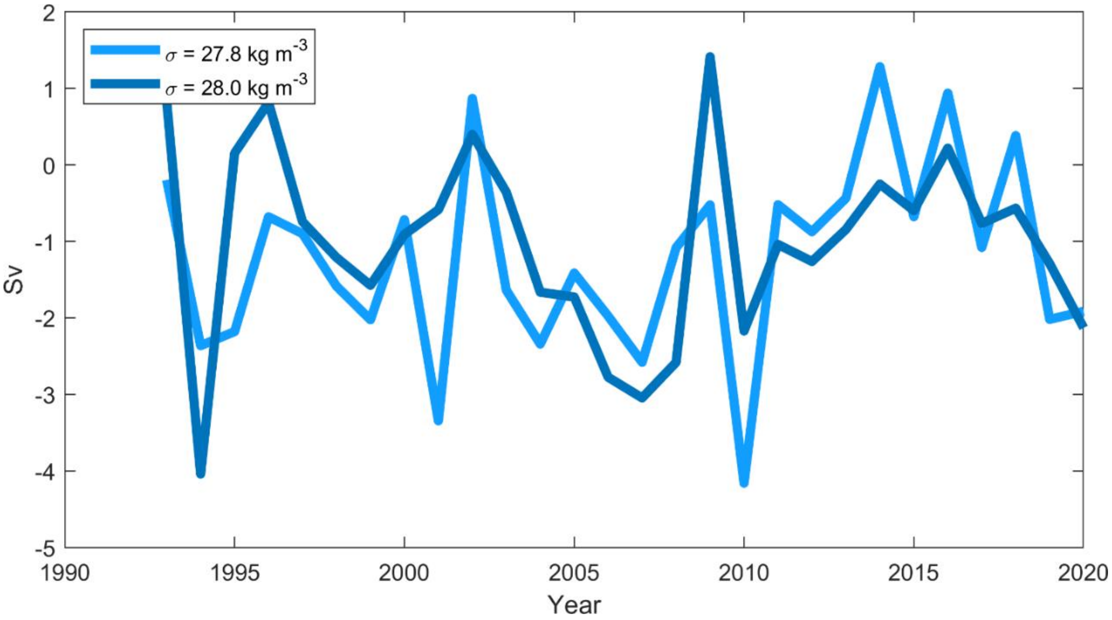

Fig. S4. Water mass transformation by diapycnal mixing. Time series of water mass transformation by diapycnal mixing (residual term; F_{res}) in the Nordic Seas at σ_o=27.8 kg m^{-3} and σ_o=28.0 kg m^{-3}.

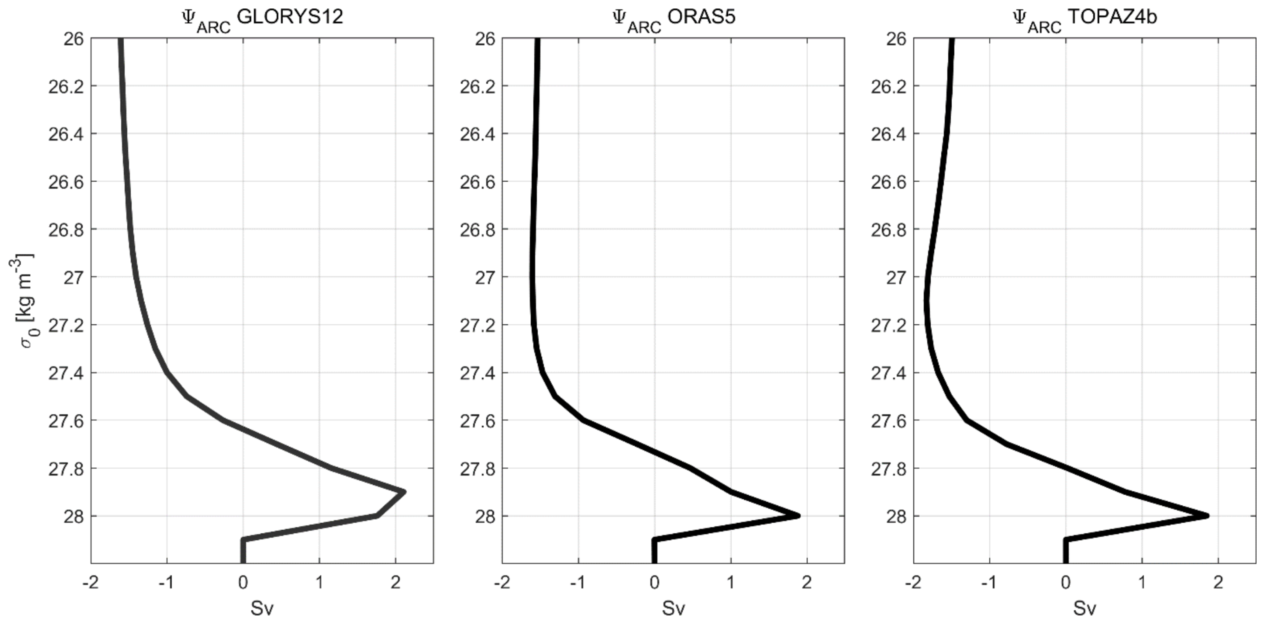

Fig. S5. Mean Arctic Ocean overturning. Time-mean (1993-2020) streamfunctions of overturning strength at the Arctic gateways (Fram Strait and BSO; Ψ_{ARC}) for GLORYS12, ORAS5 and TOPAZ4b.

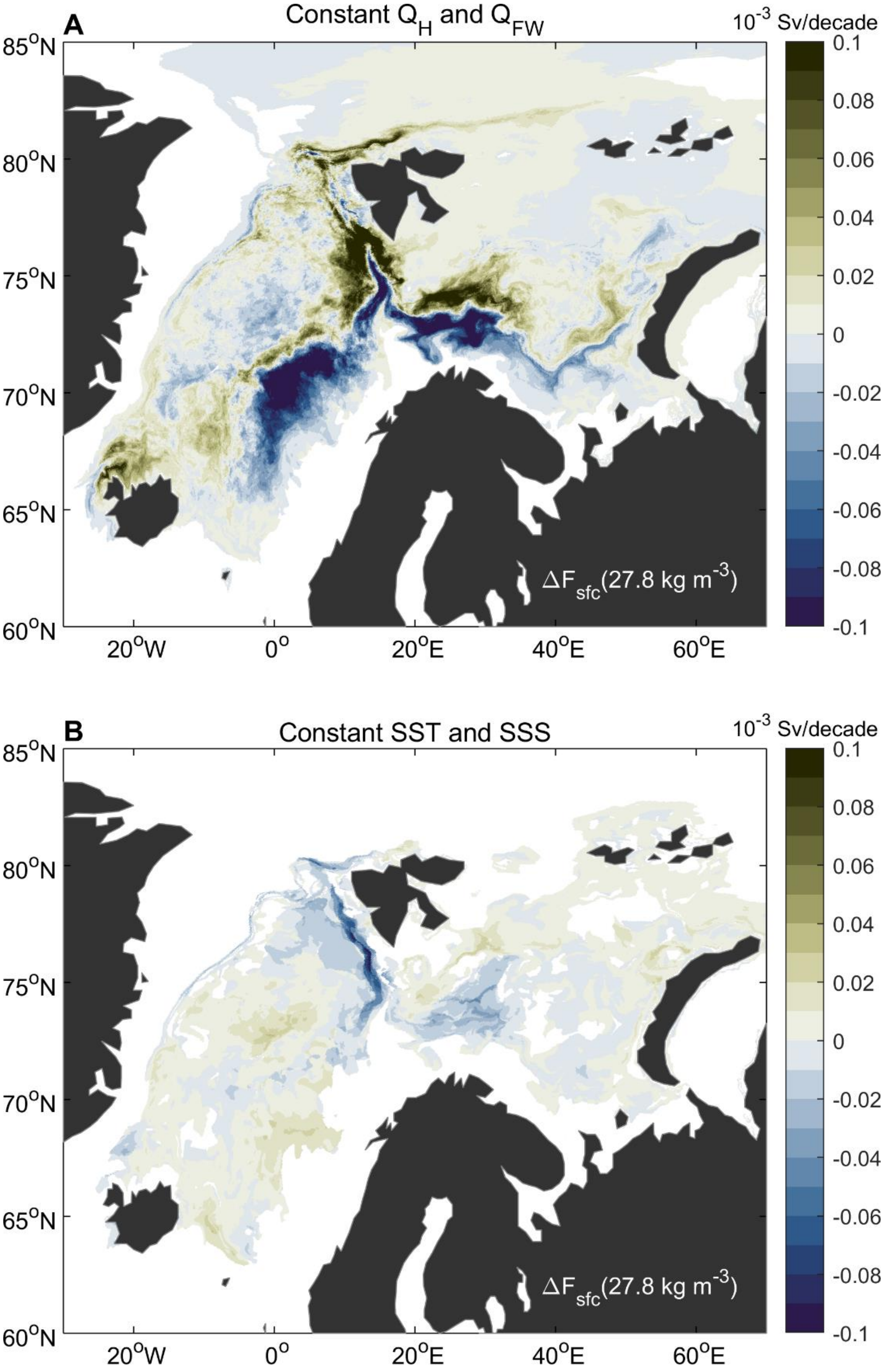

Fig. S6. Drivers of surface water mass transformation at 27.8 kg m^{-3}. Linear trend of surface-forced water mass transformation (ΔF_{sfc}) at σ_0 = 27.8 kg m^{-3} between 1993 and 2020, calculated using a climatological seasonal cycle for (**A**) surface heat and freshwater fluxes, $F_\sigma^{\mathrm{sfc}}\left(D, \overline{Q}\right)$, and (**B**) surface density, $F_\sigma^{\mathrm{sfc}}\left(\overline{D}, Q\right)$ (see Methods).

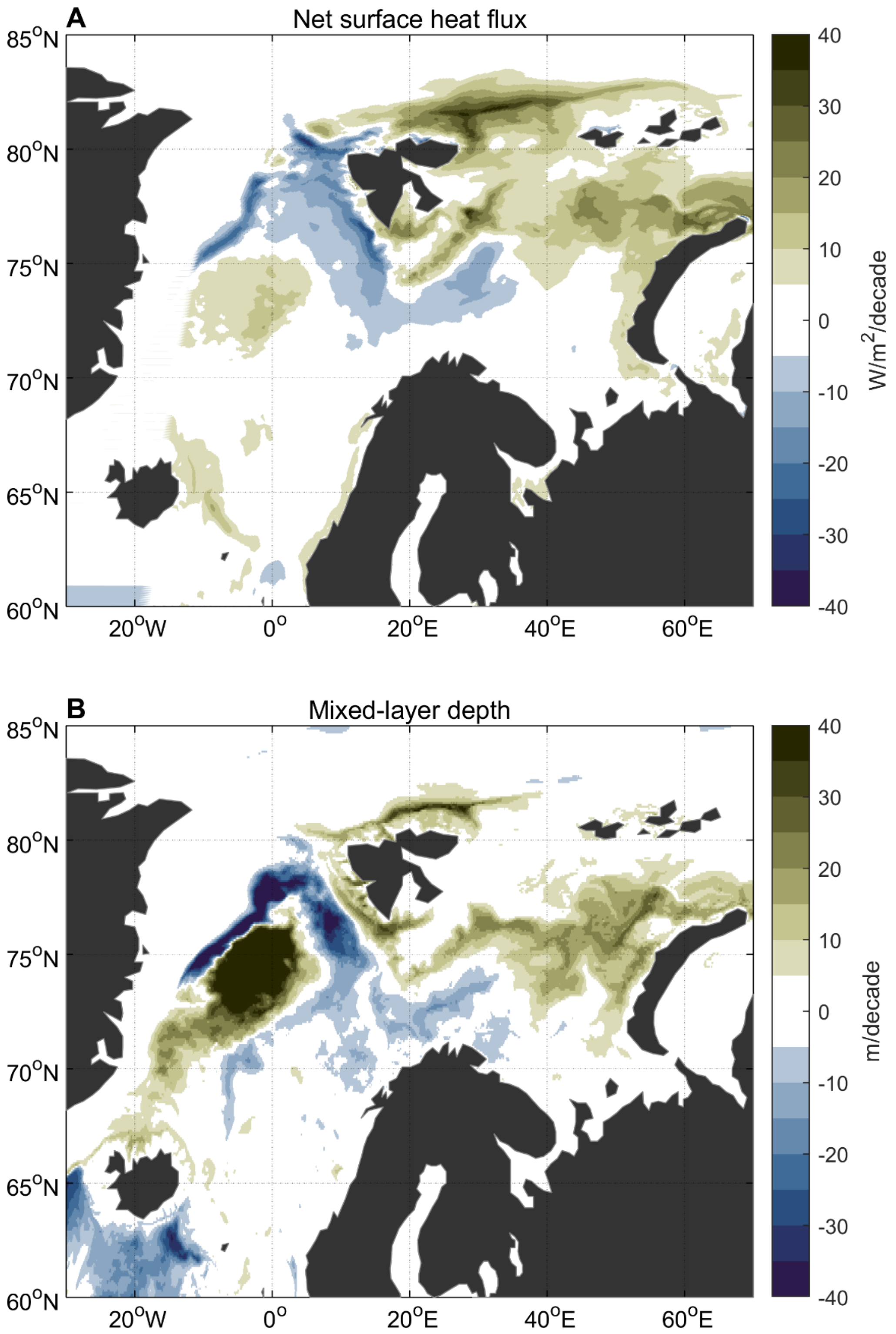

Fig. S7. Spatial pattern of trends in surface heat fluxes and mixed-layer depths. Linear trend between 1993-2020 in annual (**A**) net surface heat flux and (**B**) mixed-layer depth in GLORYS12. Positive trends in heat fluxes indicate more surface heat loss.

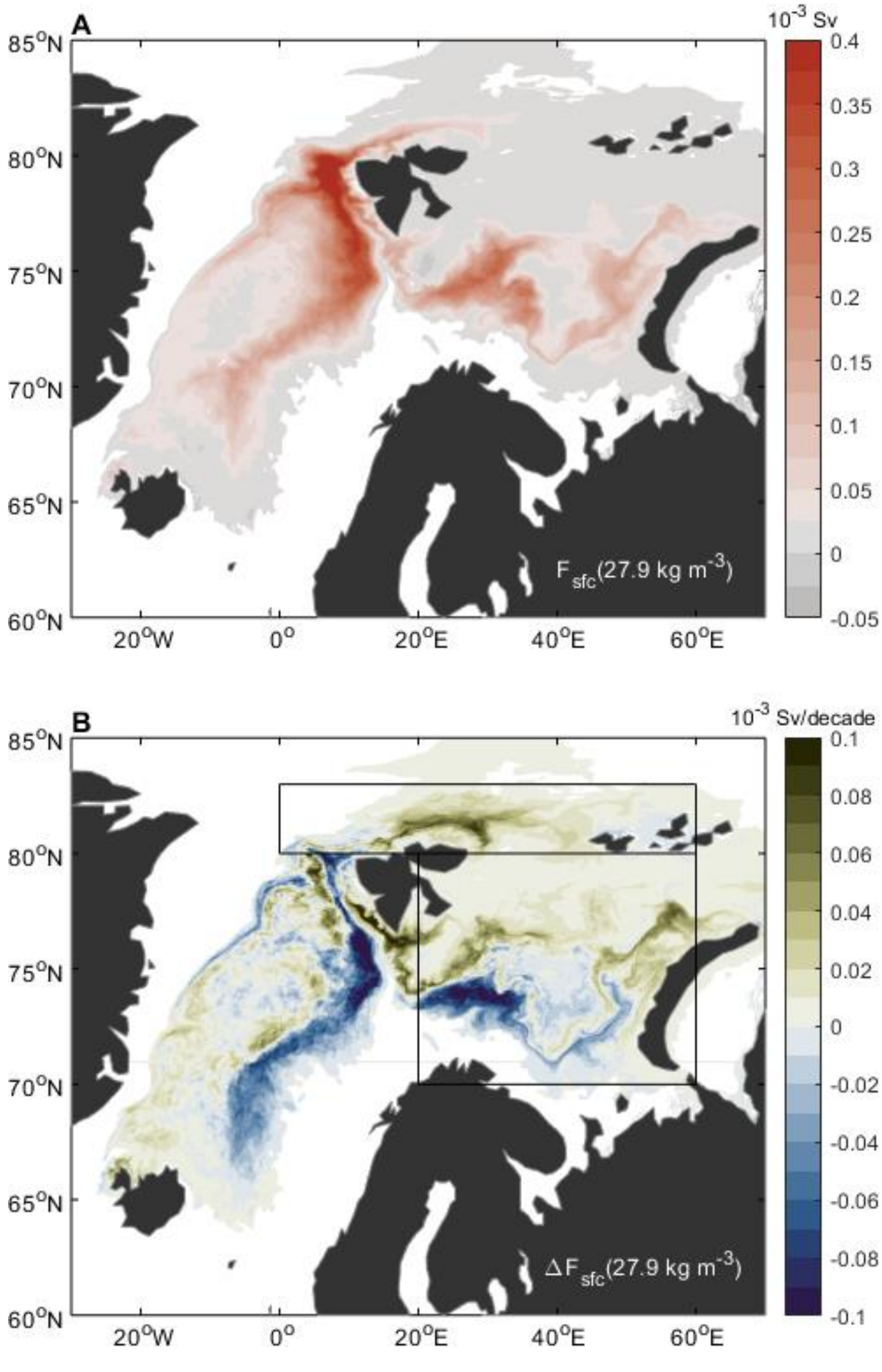

Fig. S8. Spatial pattern of surface water mass transformation at 27.9 kg m^{-3}. (**A**) Time-mean spatial pattern of surface-forced overturning (F_{sfc}) at σ_0 = 27.9 kg m^{-3} in GLORYS12. Positive values correspond to densification to this isopycnal. (**B**) Linear trend of surface-forced overturning (ΔF_{sfc}) at σ_0 = 27.9 kg m^{-3} between 1993 and 2020.

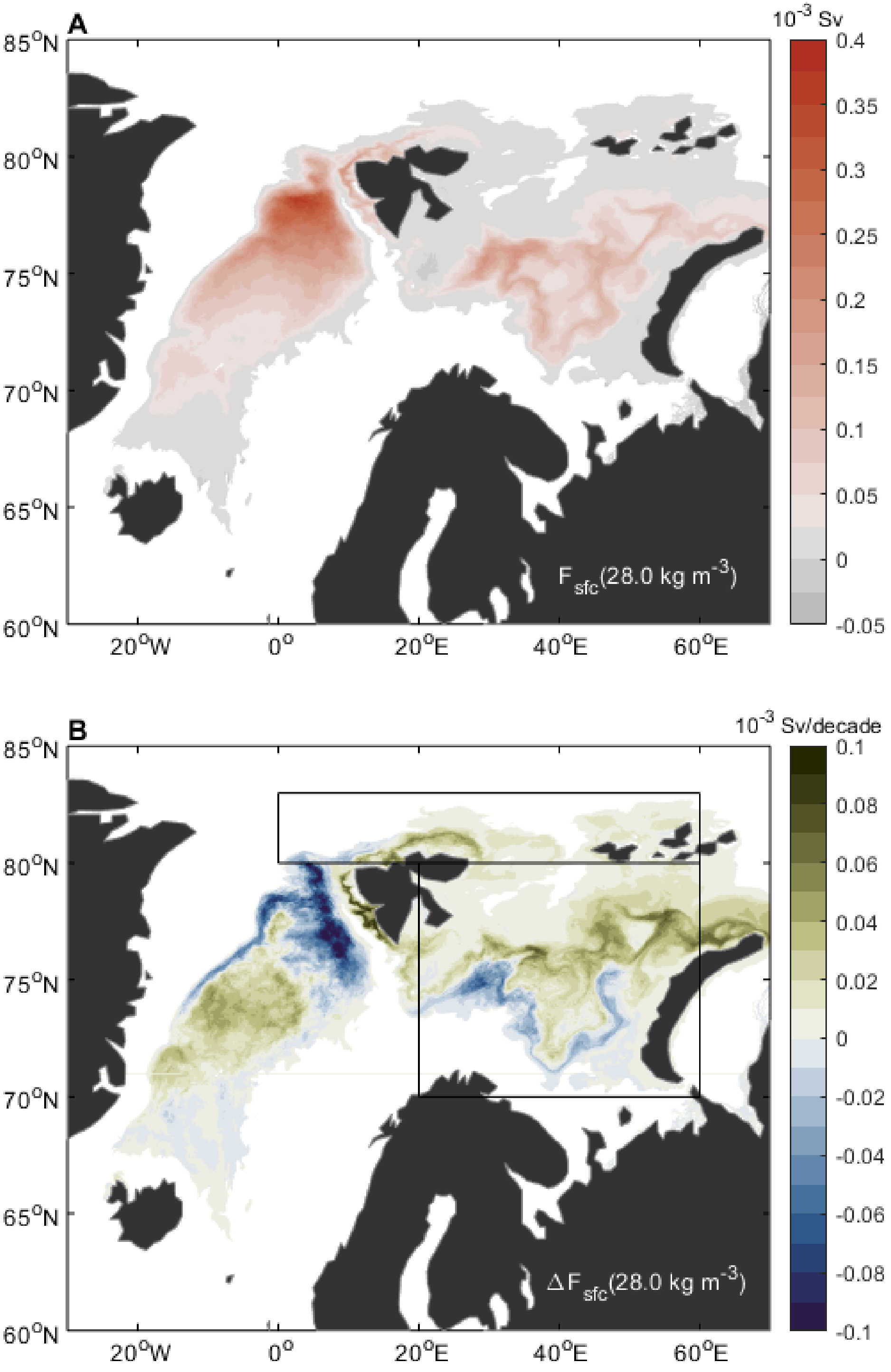

Fig. S9. Spatial pattern of surface water mass transformation at 28.0 kg m^{-3}. (**A**) Time-mean spatial pattern of surface-forced overturning (F_{sfc}) at σ_0 = 28.0 kg m^{-3} in GLORYS12. Positive values correspond to densification to this isopycnal. (**B**) Linear trend of surface-forced overturning (ΔF_{sfc}) at σ_0 = 28.0 kg m^{-3} between 1993 and 2020.

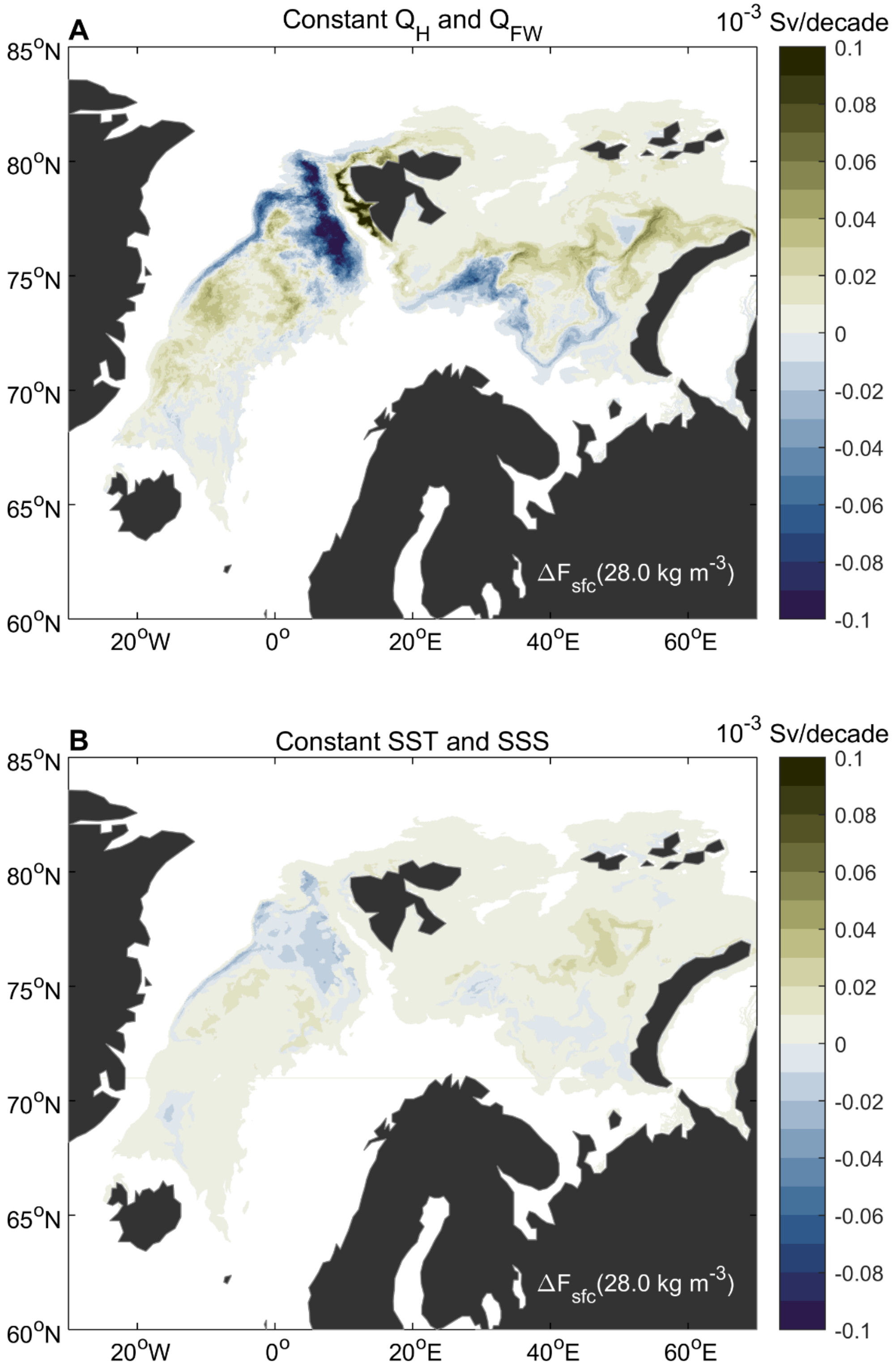

Fig. S10. Drivers of surface water mass transformation trends at 28.0 kg m^{-3}. Linear trend of surface-forced water mass transformation (ΔF_{sfc}) at σ_0 = 28.0 kg m^{-3} between 1993 and 2020, calculated using a climatological seasonal cycle for (**A**) surface heat and freshwater fluxes, $F_{\sigma}^{sfc}(D, \overline{Q})$, and (**B**) surface density, $F_{\sigma}^{sfc}(\overline{D}, Q)$ (see Methods).

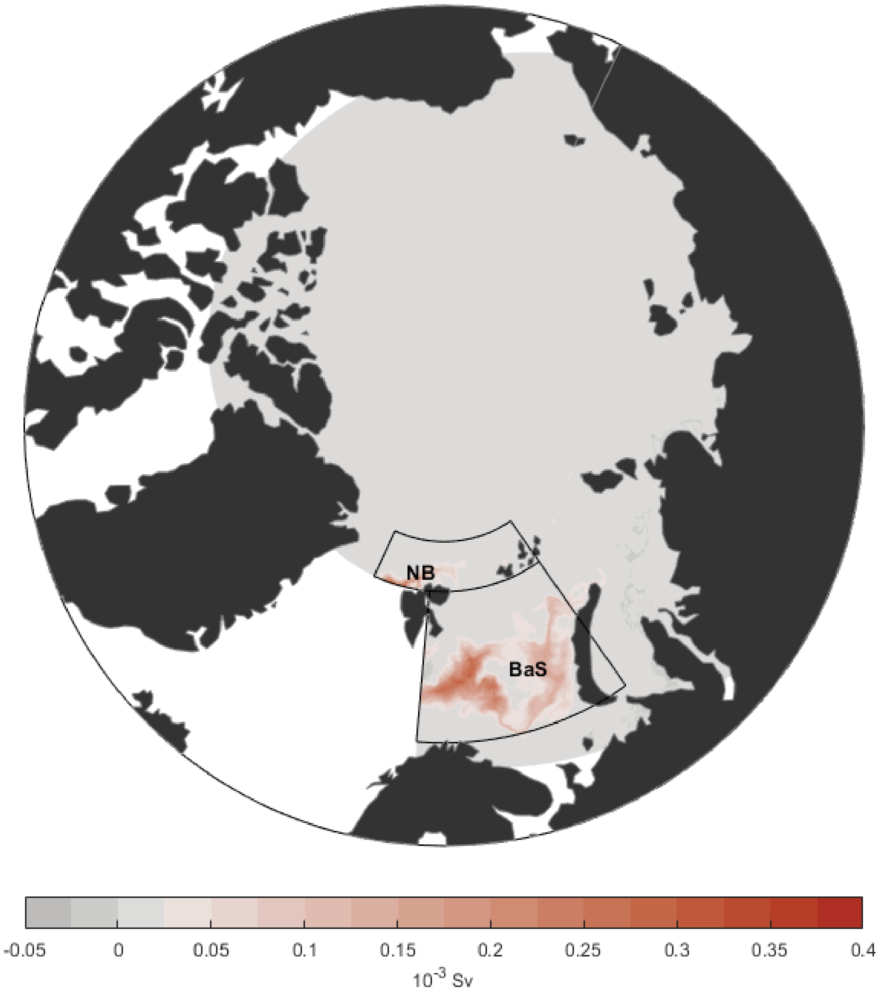

Fig. S11. Mean surface water mass transformation in the Arctic Ocean. Time-mean spatial pattern of surface-forced overturning (F_{sfc}) at σ_0 = 27.90 kg m^{-3} in the Arctic Ocean in GLORYS12. The area of the Barents Sea (BaS) and Nansen Basin (NB) are outlined.
